# Cardiac autonomic neuropathy predicts renal decline in type 1 diabetes mellitus: a 15 year follow-up study

**DOI:** 10.1007/s00125-026-06818-y

**Published:** 2026-07-29

**Authors:** Kywe Kywe Soe, Emma Robinson, Misbah Oleolo, Mohummad Goonoo, Gordon Sloan, Simon Heller, Solomon Tesfaye, Jefferson L. B. Marques, Steven Sourbron, Dinesh Selvarajah

**Affiliations:** 1 Division of Clinical Medicine, School of Medicine and Population Health, University of Sheffield, Sheffield, UK; 2 Department of Renal Medicine, Sheffield Teaching Hospitals NHS Foundation Trust, Sheffield, UK; 3 Royal Derby Hospital, Derby, UK; 4 Institute of Biomedical Engineering, Department of Electrical and Electronic Engineering, Federal University of Santa Catarina, Florianópolis, Brazil

**Keywords:** Autonomic neuropathy, Cardiac autonomic neuropathy, Diabetic kidney disease, Diabetic nephropathy

## Abstract

**Aims/hypothesis:**

The aim of this study was to determine whether cardiac autonomic neuropathy (CAN) is an independent risk factor for kidney function decline in type 1 diabetes mellitus over a 15 year follow-up period.

**Methods:**

Eighty seven participants with type 1 diabetes and eGFR >30 ml/min per 1.73 m^2^ underwent cardiovascular autonomic reflex testing at baseline. Renal data were retrieved at 4 year intervals over 15 years. Stratification of participants into Kidney Disease: Improving Global Outcomes (KDIGO) chronic kidney disease progression prognostic risk was done by using eGFR and urinary albumin/creatinine ratio (UACR). Longitudinal changes in eGFR and UACR were analysed using linear mixed-effects models with adjustment for potential confounders to determine whether CAN is an independent predictor of eGFR and UACR change over 15 years.

**Results:**

At baseline, higher KDIGO risk category was associated with increased severity of CAN (*r*=0.38, *p*<0.001) and increase in UACR (*p*<0.001) compared with the lower risk group. Participants with established CAN at baseline exhibited greater mean (SD) eGFR decline of 14.8 (21.6) ml/min per 1.73 m^2^, compared with 3.1 (7.3) and 3.3 (7.4) ml/min per 1.73 m^2^ in the early stage and those without CAN (*p*=0.004). Mean (SD) increases in UACR were 0.4 (0.9), 11.3 (30.4) and 4 (8.9) mg/mmol for no CAN, early CAN and established CAN (*p*=0.03), respectively. After adjusting for potential confounders, baseline CAN was independently associated with both accelerated eGFR decline (*p*=0.018) and greater increases in UACR (*p*=0.002) over time.

**Conclusions/interpretation:**

CAN is an independent predictor of both accelerated eGFR decline and increased albuminuria in type 1 diabetes. The study also explored the relationship between CAN and KDIGO prognostic risk in diabetic kidney disease, demonstrating that increased severity of CAN is associated with higher KDIGO prognostic risk.

**Graphical Abstract:**

**Figure:**
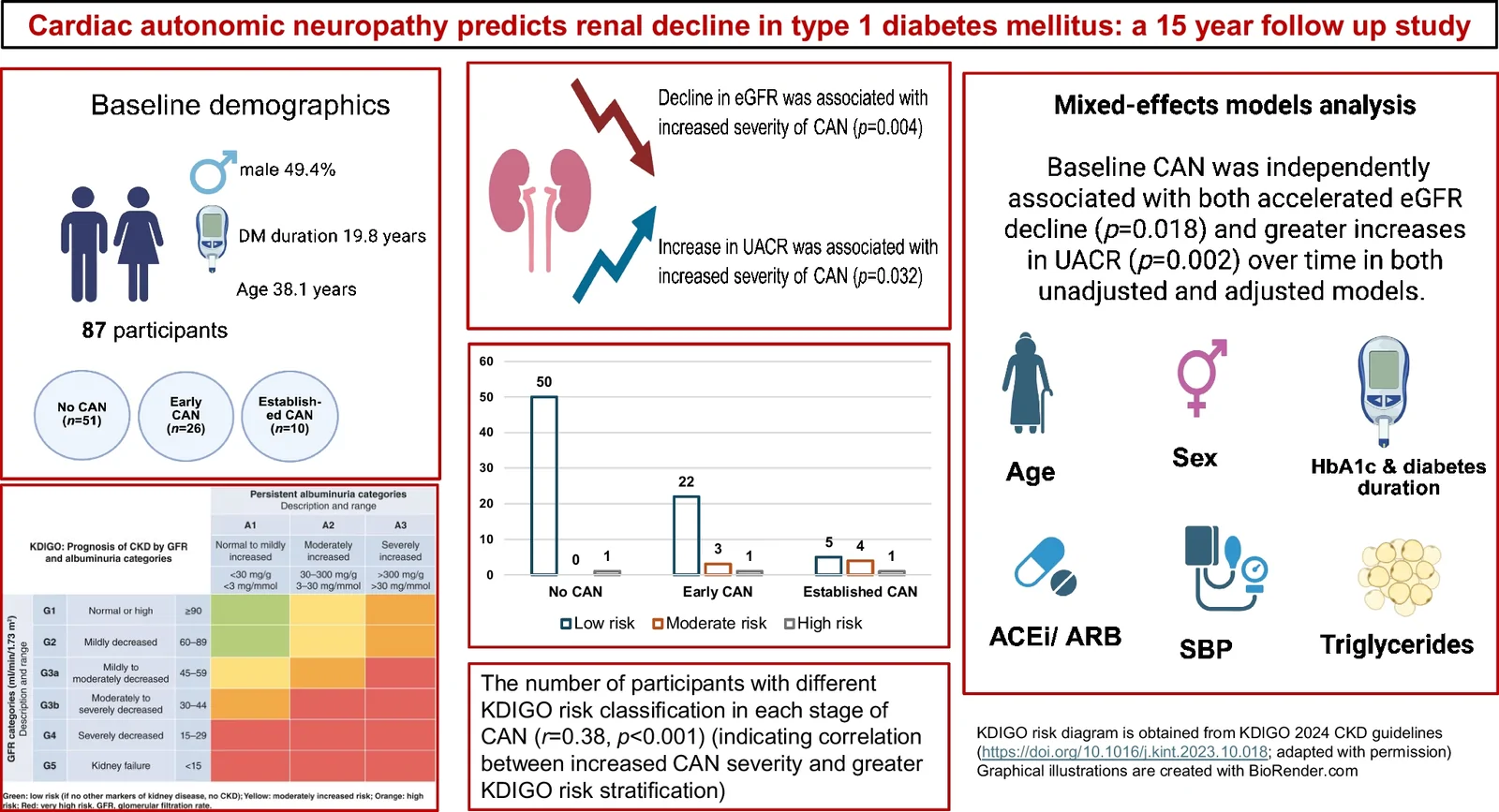

**Supplementary Information:**

The online version contains peer-reviewed but unedited supplementary material available at https://doi.org/10.1007/s00125-026-06818-y.

**Figure:**
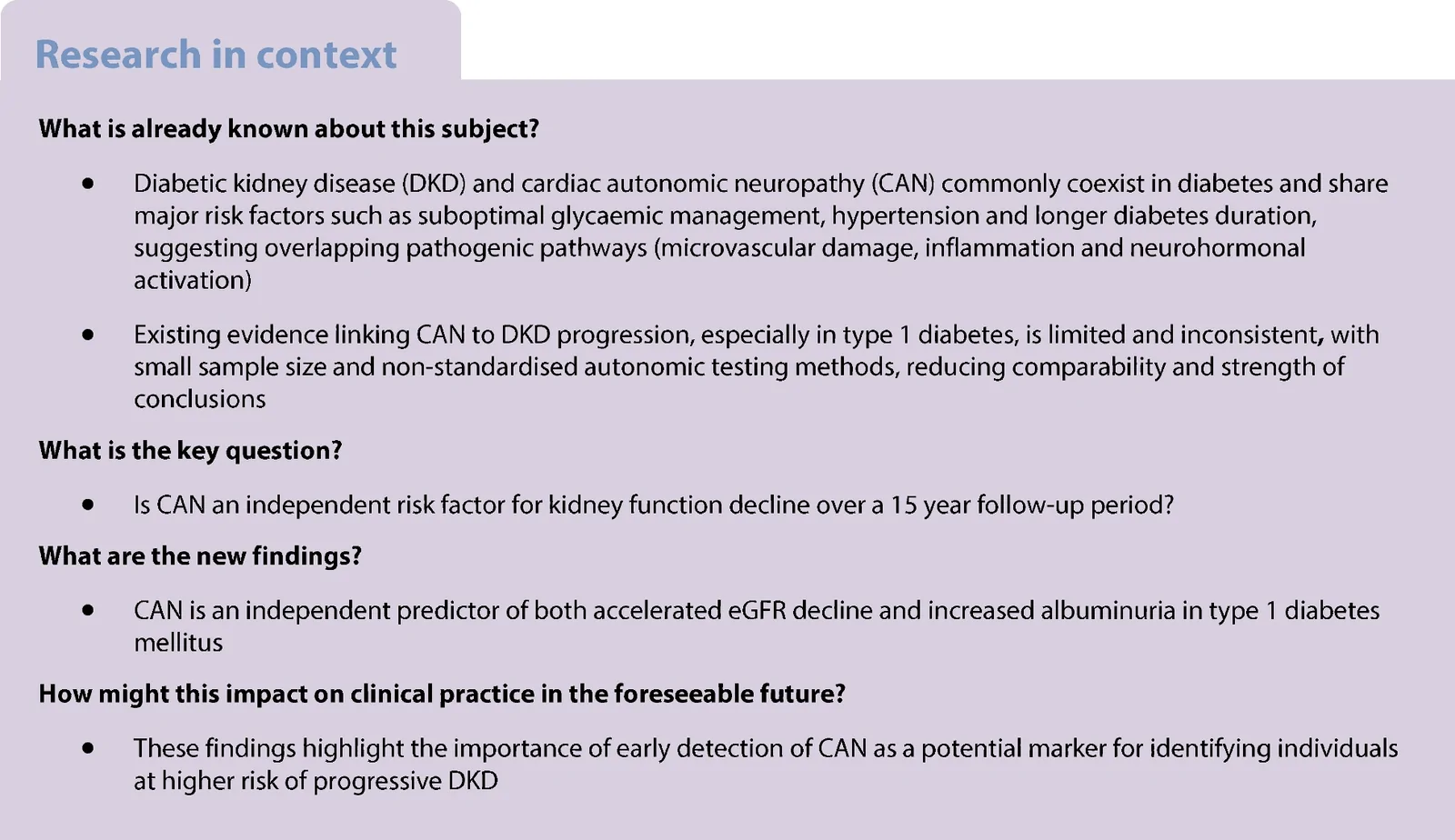

## Introduction

Diabetic kidney disease (DKD) is the leading cause of kidney failure [1] with no effective means to prevent or cure it [1]. The current treatments focus on improving glycaemic management, BP control, lifestyle modification and using reno-protective agents such as ACE inhibitors (ACEis) / angiotensin receptor blocker (ARBs). More recently, sodium–glucose cotransporter 2 inhibitors (SGLT2is) [2, 3], glucagon-like peptide-1 receptor agonists (GLP-1 RAs) [4] and mineralocorticoid receptor antagonists (MRAs) [5] have been shown to slow down the progression of DKD in type 2 diabetes mellitus. In type 1 diabetes, finerenone is shown to reduce albuminuria beyond renin–angiotensin–aldosterone system (RAAS) inhibition [6, 7], with lesser effects from SGLT2is [8–10].

Cardiac autonomic neuropathy (CAN) is often associated with DKD, sharing common risk factors and pathogenic mechanisms [11–15]. Impairment of renal autonomic function is also implicated in the progression of DKD [13, 14, 16–18]. In the early stages of CAN, imbalance in sympathetic and parasympathetic renal innervation increases intraglomerular pressure, resulting in increased sodium and albumin excretion [19–21]. As CAN progresses, increased RAAS activation further exacerbates glomerular injury and accelerates DKD progression [14, 18].

Few prospective studies [11–14] in type 1 diabetes have examined the link between CAN and DKD. Variability in study populations, sample size and inconsistent methods for assessing autonomic function limits comparability and generalisability. These differences highlight the need for larger, standardised longitudinal studies for better understanding of this relationship. One notable exception is the study by Bjerre-Christensen et al [22] which included the largest cohort of individuals with type 1 diabetes (*n*=329) with autonomic neuropathy assessed using internationally recognised indices over the longest follow-up period (mean 6.1 years). They found that sympathetic dysfunction was associated with increased albuminuria, suggesting a potential marker for DKD progression. However, a major limitation of this study was the non-random selection of participants. Control participants were selected on the basis of having longstanding normoalbuminuria despite a long history of diabetes, whereas the individuals with diabetes had established DKD with significant albuminuria at baseline. This introduces selection bias that may have carried over throughout the follow-up period. Moreover, the control group is likely subject to survivor bias (these individuals may represent a biologically resilient subgroup less prone to renal or autonomic complications), thereby underestimating the risk in the general diabetes population.

Therefore, our study aims to explore the relationship between CAN and progression of DKD in an unselected population with type 1 diabetes. We hypothesise that both presence of CAN and greater severity of CAN are associated with an accelerated decline in renal function and that CAN acts as an independent risk factor for DKD progression, beyond traditional metabolic and vascular risk factors.

## Methods

### Study population

**Fig. 1:**
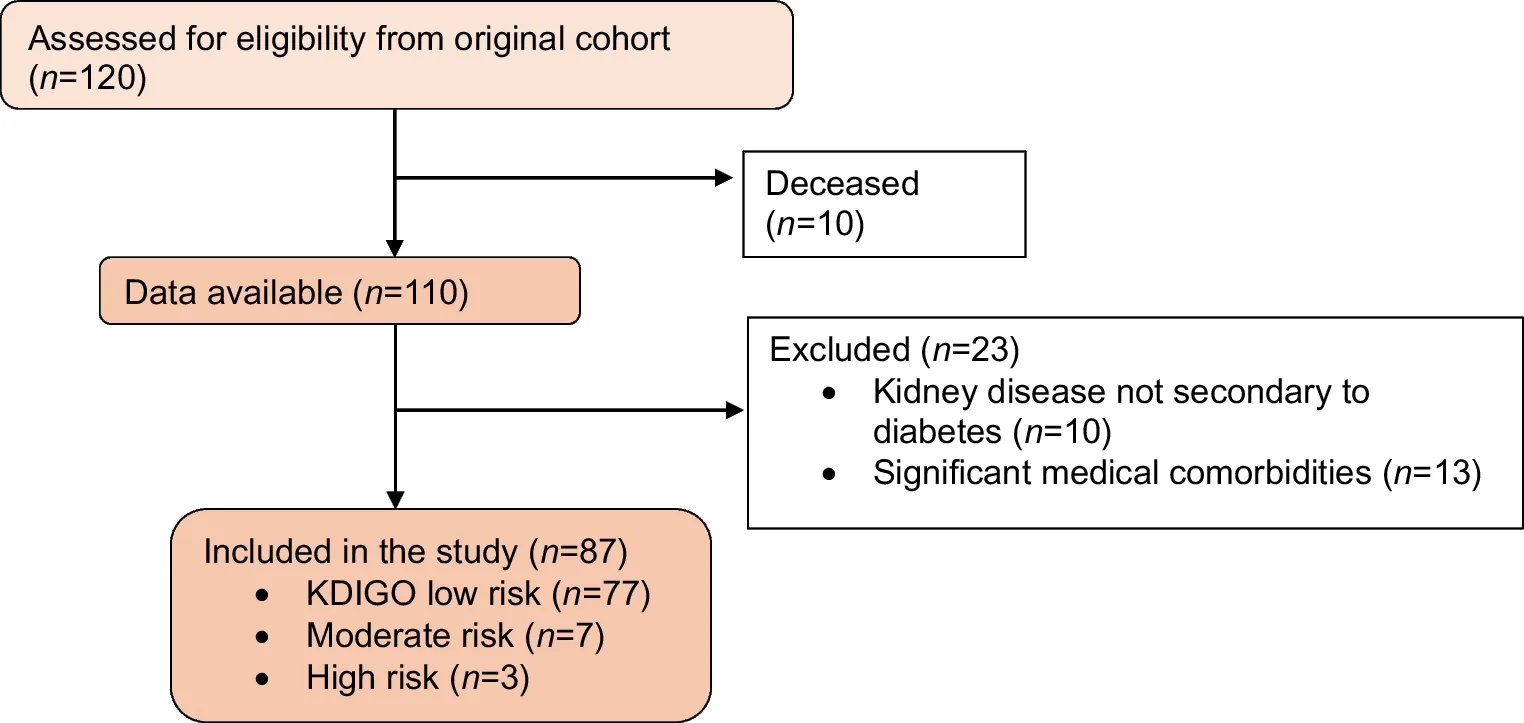
Study flow diagram

A retrospective analysis of a prospectively maintained database of 120 unselected participants with type 1 diabetes enrolled between 2007 and 2010 at the Royal Hallamshire Hospital, Sheffield, UK, was conducted. Participants were aged 18–75 years with eGFR >30 ml/min per 1.73 m^2^ (Kidney Disease: Improving Global Outcomes [KDIGO] G1–G3) and urinary albumin/creatinine ratio (UACR) <30 mg/mmol (A1 or A2) at baseline. At follow-up in 2023–2024, 110 were alive (see Fig. 1). Participants who were deceased prior to follow-up assessment (*n*=10) were excluded, as outcome data were unavailable beyond death. Cause-of-death information was not systematically recorded. After excluding those with newly diagnosed non-diabetic kidney disease or significant medical comorbidities (e.g. cancer, vasculitis, severe heart failure), 87 participants were included in the analysis. Demographic and clinical data were initially collected at baseline and updated information was collected from hospital records, primary healthcare registries and local medical records. Data on race/ethnicity were not collected in this cohort and were therefore not included in the analyses. All participants provided written informed consent, and the study had prior ethics approval.

### Assessment of CAN

At baseline visit, CAN was assessed using cardiovascular autonomic reflex tests (CARTs) according to the O’Brien’s protocol [23] (electronic supplementary material [ESM] Table 1). Participants were instructed to rest lying down for 5 min in a quiet room at a controlled temperature (18–23°C) before the examination was performed. Participants were instructed to avoid caffeine, nicotine and alcohol for 3 h prior to testing and to withhold beta-blockers on the day of assessment.

The standardised CARTs included assessment of heart rate response to deep breathing (E/I ratio), Valsalva manoeuvre, lying to standing test (30:15 ratio and BP response). Participants were divided into three groups [23].

- No CAN: all tests normal
- Early CAN: one abnormal test
- Established CAN: two or more abnormal tests

### DKD and KDIGO prognostic risk

Participants were stratified at baseline into low, moderate and high-risk prognostic groups according to the 2024 KDIGO guideline for chronic kidney disease (CKD) [24] by using eGFR and UACR. Due to the limited numbers of participants, the ‘high-risk’ and ‘very-high-risk’ categories were combined into a single 'high-risk' group. The eGFR was calculated by chronic kidney disease epidemiology collaboration (CKD-EPI) equation [25] and UACR levels were measured from an early morning urine sample [26].

### Follow-up assessments

Healthcare registries containing outcomes of the diabetes annual screening programme were used to collect endpoint data. Over the 15 year follow-up period, eGFR and UACR values were recorded at 4 year intervals: 2007–2010; 2011–2014; 2015–2018; and 2019–2022. A composite kidney outcome was defined as the first occurrence of any of the following: (1) development of severely increased albuminuria (UACR >30 mg/mmol); (2) progression to a higher KDIGO risk category; (3) a sustained ≥30% decline in eGFR from baseline; or (4) initiation of kidney replacement therapy [27]. Outcomes were assessed at the 4 yearly data collection, and the earliest occurrence was used in analyses. Death due to kidney disease was initially considered as part of the composite outcome. However, outcome data beyond death were unavailable and this component was not included in the final analyses.

### Statistical analysis

Clinical and demographic data were summarised as means with SD for continuous variables or as counts with percentages (%) for categorical variables. Data distribution was assessed using histograms. Continuous variables were compared using either Student's *t* test or the Mann–Whitney *U* test, depending on normality. Pearson’s correlation was used to analyse correlation between continuous variables.

The primary analyses aimed to examine the association between CAN stages and DKD progression, as defined by KDIGO risk groups, and to assess whether the presence and severity of CAN are linked to longitudinal changes in renal function (eGFR decline and UACR progression). These analyses are described in detail below.

At baseline, CAN stages and KDIGO risk groups were treated as categorical variables. χ^2^ tests were used to assess associations between CAN stages and KDIGO risk groups and to examine differences in categorical variables across CAN stages. Continuous variables were compared using ANOVA.

Longitudinal changes in eGFR and UACR were analysed using linear mixed-effects models to account for repeated measurements within individuals over time. Time was modelled as a continuous variable, such that the estimated coefficient for time represented the average rate of change over follow-up. Because UACR was positively skewed, log-transformed UACR values were used in longitudinal models. All models included CAN status, time, and a time-by-CAN interaction term to assess differences in trajectories between groups. Random intercepts were included for participants to account for within-subject correlation.

Sequential models were fitted with covariates as follows:

- Model 1: unadjusted
- Model 2: adjusted for age and sex
- Model 3: adjusted for age, sex, diabetes duration, HbA_1c_, systolic BP, triglycerides and ACEi/ARB use

Covariates were selected based on their established associations with both CAN and DKD progression. These factors are known confounders in renal and autonomic dysfunction studies and were chosen to improve model precision and account for key demographic and clinical characteristics that may influence renal outcomes.

Secondary analyses were conducted to assess participants with normal eGFR (>90 ml/min per 1.73 m^2^, G1) and normoalbuminuria (UACR <3 mg/mmol, A1). The goal was to determine whether the presence of CAN significantly impacts this subset of participants who had normal renal function at baseline. Given their optimal renal function and absence of albuminuria, the aim was to explore whether CAN could exert a similar detrimental effect on renal outcomes as seen in participants with poorer baseline function.

The composite renal outcome was analysed as a binary outcome at 15 years of follow-up. Logistic regression models were used to evaluate the association between CAN status and the presence of the composite renal outcome. The dependent variable was the occurrence of the composite outcome (yes/no), and independent variables included CAN status and baseline characteristics (diabetes duration, HbA_1c_, systolic BP and ACEi/ARB use). Sequential models were fitted, including unadjusted and adjusted analyses. ORs with 95% CIs were reported. Given that outcomes were assessed at discrete study visits, time-to-event analyses were not performed. All analyses were conducted using SPSS (version 29.0), with statistical significance set at *p*<0.05.

## Results

### Baseline demographics

Complete baseline visit and follow-up data were available for 87 participants (Table 1). At baseline, 49.4% (*n*=43) were men, with a mean ± SD age of 38.1±10.6 years and diabetes duration of 19.8±11.5 years. Mean ± SD eGFR was 88.4±8.7 ml/min per 1.73 m^2^ (87.3% G1, 9.1% G2, 3.4% G3) and mean ± SD UACR was 1.8±4.3 mg/mmol (90.8% A1, 8% A2, 1.1% A3). Additionally, 19.5% of participants had hypertension. Participants with established CAN were older (ANOVA *p*=0.005) and had a longer duration of diabetes (ANOVA *p*=0.06). Baseline eGFR was significantly lower in established CAN (ANOVA *p*<0.001). Other variables, including HbA_1c_, systolic and diastolic BP, cholesterol, triglycerides and medication use, showed no statistically significant differences among the groups. While the prevalence of retinopathy and history of hypertension were higher in the established CAN group, these differences were not statistically significant.

**Table 1:** Baseline demographic data of participants with different CAN stages

Characteristic	Overall (*N*=87)	Normal/no CAN (*n*=51)	Early CAN (*n*=26)	Established CAN (*n*=10)	*p* value
Age at baseline, years	38.1±10.6	38.8±8.1	33.6±12.8	46±11	0.005
Men, *n* (%)	43 (49.4)	27 (52.9)	12 (46.1)	4 (40)	0.70
Diabetes duration, years	19.8±11.5	18.1±9.9	19.9±13.5	27.6±11.4	0.06
eGFR, ml/min per 1.73 m^2^					
eGFR at baseline	88.4±8.7	90.2±2.7	88.6±8.8	78.8±18.7	<0.001
eGFR decline	4.6±10.5	3.3±7.4	3.1±7.3	14.8±21.6	0.004
eGFR at 15 years	84±14.4	87.2±7.8	85.4±15.1	64.8±22.8	<0.001
UACR, mg/mmol					
UACR at baseline	1.8±4.3	1.4±4.6	1.5±2	4.9±6.3	0.06
UACR, median (IQR)	0.70 (0.40–1.20)	0.70 (0.4–1.05)	0.8 (0.45–1.75)	1.1 (0.65–9.03)	
UACR increase	4.1 (17.4)	0.4 (0.9)	11.3 (30.4)	4 (8.9)	0.03
UACR, median (IQR)	0.0 (0.0–1.0)	0.0 (0.0–0.6)	0.75 (0.0–3.1)	0.0 (0.0–2.1)	
UACR at 15 years	5.9±19.6	1.4±3.2	15.3±35.9	8.6±11.4	0.02
UACR, median (IQR)	1.0 (0.50–2.35)	1.0 (0.40–1.25)	1.5 (0.60–4.23)	4.0 (0.98–11.68)	
HbA_1c_, mmol/mol	69.69±14.7	67.9±14.5	70.7±12.5	77±19.7	0.2
HbA_1c_, %	6.6±1.3	6.4±1.3	6.7±1.1	7.2±1.8	
Systolic BP, mmHg	125.9±14.9	122.8±12.5	129.1±16.2	133.4±19.2	0.05
Diastolic BP, mmHg	73.68±7.7	73±7.2	75.5±9.2	72±5.7	0.32
Total cholesterol, mmol/l	4.4±0.86	4.4±0.87	4.5±0.81	4.3±0.99	0.77
Triglyceride, mmol/l	1.2±0.63	1.17±0.63	1.29±0.69	1.32±0.51	0.70
Use of ACEI/ARB, *n* (%)	31 (35.6)	15 (29.4)	10 (38.5)	6 (60)	0.89^a^
Use of β-blocker, *n* (%)	5 (5.7)	1 (2)	3 (11.5)	1 (10)	0.193^a^
Use of statin, *n* (%)	64 (73.6)	39 (76.4)	16 (61.5)	9 (90)	0.17^a^
Diabetic retinopathy, *n* (%)	66 (75.9)	36 (70.5)	21 (80.7)	9 (90)	0.67^a^
History of hypertension (%)	17 (19.5)	7 (13.7)	5 (19.2)	5 (50)	0.15^a^

Data are shown as mean ± SD, *n* (%) or median (IQR)

^a^χ^2^ test

### Fifteen year follow-up data in different CAN stages

At the 15 year follow-up, mean ± SD eGFR values were lowest in the established CAN group (64.8±22.8 ml/min per 1.73 m^2^) than in the normal (87.2±7.8 ml/min per 1.73 m^2^) and early CAN (85.4±15.1 ml/min per 1.73 m^2^, ANOVA *p*<0.001) groups. A more pronounced decline in eGFR was associated with increased severity of CAN (*p*=0.004). A similar trend was observed in the progression of urinary albuminuria (*p*=0.03), with participants in the early and established CAN groups exhibiting higher baseline and follow-up UACR values (Table 1).

### CAN and DKD progression

Longitudinal changes in eGFR and UACR were analysed using mixed-effects models. In fully adjusted models, eGFR declined significantly over time (−0.66 ml/min per 1.73 m^2^ per year; *p*<0.001). There was a significant interaction between time and CAN status (*p*=0.018), indicating a faster decline in participants with CAN than in those without CAN (−0.66 vs −0.30 ml/min per 1.73 m^2^ per year). Baseline eGFR did not differ significantly between the two groups (*p*=0.223). Increasing age and systolic BP were independently associated with lower eGFR (*p*<0.05), while other covariates were not significant (Table 2).

**Table 2:** Mixed-effects models for longitudinal changes in eGFR

Variable	Model 1^a^		Model 2^b^		Model 3^c^	
	β (SE)	*p* value	β (SE)	*p* value	β (SE)	*p* value
Time	−0.552 (0.102)	<0.001	−0.659 (0.117)	<0.001	−0.659 (0.118)	<0.001
CAN	3.877 (2.253)	0.088	4.280 (1.946)	0.030	2.482 (2.022)	0.223
Time × CAN	0.272 (0.135)	0.044	0.358 (0.151)	0.019	0.360 (0.151)	0.018
Age	–	–	−0.595 (0.079)	<0.001	−0.553 (0.093)	<0.001
Sex	–	–	−0.706 (1.691)	0.678	−0.066 (1.747)	0.970
Diabetes duration	–	–	–	–	−0.101 (0.087)	0.251
Triglycerides	–	–	–	–	−0.781 (1.373)	0.571
HbA_1c_	–	–	–	–	−0.083 (0.058)	0.155
Systolic BP	–	–	–	–	−0.132 (0.062)	0.037
ACEi/ARB	–	–	–	–	2.310 (1.887)	0.225

β coefficients (SE) from linear mixed-effects models are shown

^a^Model 1: unadjusted

^b^Model 2: adjusted for age and sex

^c^Model 3: adjusted for age, sex, diabetes duration, HbA_1c_, systolic BP, triglycerides and ACEi/ARB use

Similarly, log-transformed UACR increased significantly over time (*p*<0.001). There was a significant time × CAN interaction (*p*=0.002), indicating a substantially greater increase in UACR among participants with CAN. Specifically, UACR increased by approximately 121% per time unit in the CAN group, whereas minimal change (~4%) was observed in those without CAN. Baseline UACR did not differ significantly between the two groups (*p*=0.924). Age was independently associated with higher UACR over time (*p*=0.024), while other covariates were not significant (Table 3).

**Table 3:** Mixed-effects models for longitudinal changes in UACR

Variable	Model 1^a^		Model 2^b^		Model 3^c^	
	β (SE)	*p* value	β (SE)	*p* value	β (SE)	*p* value
Time	0.642 (0.162)	<0.001	0.791 (0.183)	<0.001	0.791 (0.183)	<0.001
CAN	−0.674 (2.003)	0.737	−0.473 (2.048)	0.818	−0.206 (2.168)	0.924
Time × CAN	−0.618 (0.213)	0.004	−0.757 (0.236)	0.002	−0.753 (0.236)	0.002
Age	–	–	0.197 (0.068)	0.005	0.196 (0.085)	0.024
Sex	–	–	−1.392 (1.466)	0.345	−1.776 (1.593)	0.269
Diabetes duration	–	–	–	–	−0.021 (0.080)	0.790
Triglycerides	–	–	–	–	1.134 (1.251)	0.368
HbA_1c_	–	–	–	–	−0.002 (0.053)	0.966
Systolic BP	–	–	–	–	0.007 (0.057)	0.898
ACEi/ARB	–	–	–	–	1.164 (1.719)	0.501

β coefficients (SE) from linear mixed-effects models are shown

^a^Model 1: unadjusted

^b^Model 2: adjusted for age and sex

^c^Model 3: adjusted for age, sex, diabetes duration, HbA_1c_, systolic BP, triglycerides and ACEi/ARB use

### CAN and renal function decline in participants classified as G1A1

A more detailed analysis was conducted to assess the extent of decline in eGFR and increase in UACR among participants who initially had an eGFR ≥90 ml/min per 1.73 m^2^ (classified as G1) and normoalbuminuria (UACR <3 mg/mmol) (classified as A1). The analysis showed that the CAN group had a mean ± SD UACR increase of 7.0±20.0 mg/mmol, compared with 0.4±0.9 mg/mmol in the No CAN group in the unadjusted model (model 1, *p*=0.03; 95% CI 0.7, 12.4) (ESM Table 2). However, the difference was no longer significant after adjusting for age and sex (model 2, *p*=0.09; 95% CI −0.8, 12.5). The correlation regained significance when additional factors such as diabetes duration, HbA_1c_, systolic BP and use of ACEis or ARBs were included into the model (model 3, *p*=0.026; 95% CI 1.0,15.5). In contrast, for eGFR, no significant correlation for eGFR was observed in any of the three models, and the extent of eGFR decline remained comparable between the groups.

### KDIGO risk classification and CAN

**Fig. 2:**
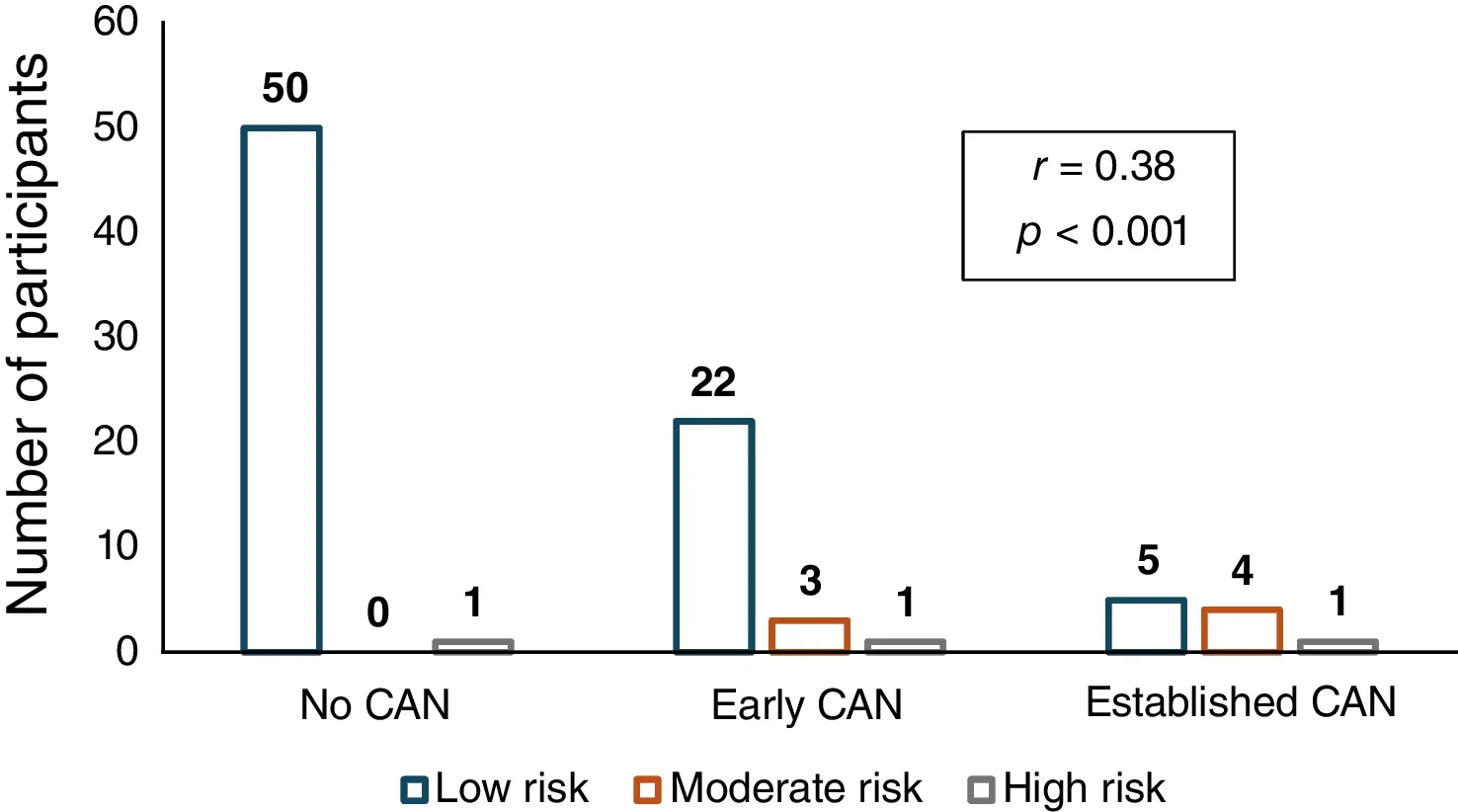
The numbers of participants with different KDIGO risk classification in each stage of CAN. The *r* and* p* values indicate a direct correlation between stage of CAN with higher prognostic risk in KDIGO risk stratification

The distribution of KDIGO risk categories among participants with varying stages of CAN showed a clear concentration of low-risk individuals in the No CAN group, with a progressive shift towards higher risk categories as the CAN severity increased (*r*=0.38, *p*<0.001) (Fig. 2). Notably, the established CAN group had a higher proportion of moderate-risk individuals compared with the other groups, indicating a correlation between increased CAN severity and greater risk classification.

In the KDIGO risk assessment, a high proportion of participants were classified as low prognostic risk (88.5%, *n*=77), followed by moderate risk (8%, *n*=7) and high risk (3.5%, *n*=3) for CKD progression (ESM Table 3). The analysis showed no significant difference in eGFR decline across KDIGO risk groups (*p*=0.30). However, UACR increased significantly in the high/very high-risk group (*p*<0.001).

### Renal endpoint assessment and CAN

A significantly greater proportion of participants with CAN (30.6%, *n*=11) reached the composite renal outcome at 15 years compared with those without CAN (11.8%, *n*=6; *p*=0.03). In logistic regression analysis, CAN status was the only independent predictor of the composite renal outcome after variable selection (β [SE] 1.26 [0.57]; Wald χ^2^ 4.86; *p*=0.03). Participants with CAN had 3.5-fold higher odds of developing the composite renal outcome compared with those without CAN (OR [95% CI] 3.5 [1.1, 10.7]; *p*=0.03).

The number of events for each component of the composite renal outcome was as follows: development of severely increased albuminuria (UACR >30 mg/mmol), *n*=3; progression to a higher KDIGO risk category, *n*=7; and ≥30% decline in eGFR, *n*=5.

No participants were started on kidney replacement therapy during follow-up.

## Discussion

In this 15 year follow-up study of an unselected cohort of adults with type 1 diabetes, greater baseline CAN severity was independently associated with both accelerated decline in eGFR and a marked increase in UACR over time. These associations remained significant after adjustment for age, sex, diabetes duration, HbA_1c_, systolic BP, triglycerides and ACEi/ARB therapy.

Participants with CAN exhibited a faster rate of eGFR decline than those without CAN, alongside a substantially greater progression of albuminuria, whereas baseline renal function did not differ between two groups. Among participants with preserved renal function at baseline (G1A1), CAN was not associated with eGFR decline but remained independently associated with progression of UACR following full covariate adjustment. These findings are consistent with prior reports linking CAN to increased albuminuria and extend existing evidence by demonstrating that CAN is also associated with accelerated decline in kidney function over time [19, 22, 28–30].

A key strength of this study is the use of an unselected, population-based cohort of adults with type 1 diabetes, enhancing the representativeness and generalisability of our findings to the broader clinical population. At baseline, the prevalence of CAN was 41%, consistent with previously reported estimates in cohorts with type 1 diabetes [31]. Participants with established CAN were older, had a longer duration of diabetes and lower eGFR than those with no CAN or early CAN. No statistically significant differences were observed across groups in HbA_1c_, systolic or diastolic BP, retinopathy or hypertension prevalence, lipid profiles, or reno-protective medication use such as ACEi/ARB. Consistent with their more advanced clinical phenotype, participants with CAN were more frequently stratified into higher KDIGO CKD risk categories at study entry.

Over the 15 year follow-up, the presence and severity of CAN were significantly associated with an increased risk of adverse renal outcomes, including progression to severely increased albuminuria (UACR >30 mg/mmol), transition to a higher KDIGO risk category and ≥30% decline in eGFR. These associations reinforce CAN as a clinically meaningful marker of heightened renal risk. However, the associations observed may reflect shared underlying microvascular pathology common to multiple diabetes complications rather than a strictly causal relationship between CAN and kidney function decline.

Several biological mechanisms have been proposed to explain the link between CAN and DKD. Early parasympathetic dysfunction leading to relative sympathetic overactivity is thought to contribute to systolic hypertension, resting tachycardia and increased cardiac output, haemodynamic changes that promote glomerular membrane injury through excessive filtration of sodium and albumin [13, 16, 32, 33]. Impaired parasympathetic regulation may also lead to a loss of nocturnal BP dipping, resulting in higher nocturnal intraglomerular pressure and increased sodium and albumin excretion [11, 19–21, 34]. In addition, relative increase in sympathetic tone as a result of early parasympathetic denervation may also activate the intrarenal RAAS, leading to renal vasoconstriction, ischaemia and structural injury, including glomerular basement membrane thickening and mesangial matrix expansion, thereby accelerating DKD progression [9, 31].

Autonomic dysfunction has additionally been linked to increased inflammation and oxidative stress. Impairment of the sympathetic nervous system may disrupt the activity of inflammatory cytokines and chemokines [15, 35], several of which have been identified as potential therapeutic targets and biomarkers for DKD [15, 36]. Oxidative stress may be further amplified by impaired antioxidant defence mechanisms, including reduced activity of enzymatic scavengers and increased production of reactive oxygen species, contributing to progressive renal injury [34, 35]. In advanced stages of CAN, sympathetic denervation may exacerbate renal dysfunction by impairing sodium handling, reducing renal blood flow and promoting vasoconstriction, ultimately leading to a decline in eGFR [11, 12].

The absence of a nocturnal decline in both systolic and diastolic BP among individuals with CAN has been consistently reported in multiple studies using 24 h ambulatory BP monitoring [19–21, 37–39]. In contrast, evidence for other neuropathic mechanisms affecting renal vascular haemodynamic remains limited. Future research employing advanced imaging techniques, such as MRI, is needed to better characterise renal haemodynamic alterations in this context [40].

Our findings suggest that the influence of CAN on DKD progression may be dependent on when CAN develops in the course of kidney disease. Specifically, the presence of CAN prior to the onset of DKD appeared to have a minimal impact on subsequent eGFR decline. In contrast, CAN occurring in the setting of established or more advanced DKD may act as an accelerator of renal function decline. However, due to the relatively small number of participants with advanced DKD at baseline, we were unable to formally test this temporal interaction. Larger studies with sufficient representation across DKD stages are needed to determine whether CAN contributes causally to kidney disease progression and to clarify its role as a potential therapeutic target.

Evidence from the ADDITION study demonstrates that early identification and management of cardiometabolic risk factors in individuals with or at risk of cardiovascular disease [41] can significantly reduce all-cause mortality, emphasising the value of timely intervention. Within this context, our findings highlight autonomic dysfunction, specifically CAN, as a potential early marker and contributor to DKD progression. This reinforces the rationale for routine, systematic screening for CAN in clinical practice. Although the ADA currently recommends CAN screening, implementation remains inconsistent. Incorporating regular CAN assessment into diabetes care pathways could facilitate earlier identification of high-risk individuals, support more targeted risk-reduction strategies, and ultimately improve long-term renal outcomes.

Strengths of this study include the recruitment of participants with relatively preserved renal function (eGFR >30 ml/min per 1.73 m^2^), with most participants exhibiting normal renal parameters at baseline (eGFR >90 ml/min per 1.73 m^2^ and normoalbuminuria). This facilitated the examination of early predictors of renal decline over a 15 year follow-up period. Longitudinal data collection at 4 year intervals enabled robust analysis of renal endpoints, and the low attrition rate enhanced the validity of our findings. Crucially, all participants underwent standardised gold-standard assessments for CAN, ensuring consistency in the assessment of autonomic function across the cohort.

Limitations of this study include its single-centre design and the relatively small number of participants with lower baseline eGFR or established albuminuria, which limited subgroup analyses of moderate- and high-risk individuals. Additionally, blood samples were collected at 4 year intervals during the 15 year follow-up. While this allowed for longitudinal assessment, more frequent sampling, such as annual measurements, would have enabled a more precise determination of the timing and trajectory of changes in eGFR and UACR. While the sample size may limit detection of small effects, it was sufficient to identify clinically meaningful differences in renal outcomes and to explore associations across CAN severity stages. These findings provide a foundation for hypothesis generation and inform the design of future, larger-scale prospective studies. Another potential limitation of this study is the exclusion of mortality from the composite renal endpoint. Participants who had died before follow-up assessment were excluded, and cause-of-death data were not available. Consequently, some individuals with more advanced disease may have died from renal or competing non-renal causes before reaching recorded renal endpoints. This may introduce survivorship bias and could have led to underestimation of the true burden of progressive kidney disease.

Additionally, medications are a recognised cause of altered autonomic responses and may contribute to false-positive CART findings. Agents most commonly implicated include cardiovascular drugs (e.g. diuretics, nitrates, β-blockers and α-blockers) and non-cardiovascular drugs (e.g. antidepressants, antipsychotics and benzodiazepines) [42]. In this study, participants were instructed to withhold β-blockers on the day of testing; however, the potential influence of other medications cannot be entirely excluded. A further limitation is that individual CART components were not available for separate analysis; therefore, we were unable to determine whether specific parasympathetic or sympathetic abnormalities were differentially associated with renal outcomes. Future studies examining individual autonomic test measures may provide greater mechanistic insight into the relationship between autonomic dysfunction and DKD.

Despite its limitations, this study provides novel evidence supporting a potential role for CAN as an independent risk factor for renal decline in individuals with type 1 diabetes. To our knowledge, it is among the first to examine the relationship between CAN and KDIGO prognostic risk categories in the context of DKD. We demonstrated that greater CAN severity has significant association with higher KDIGO CKD risk and that CAN predicts renal function decline independently of traditional risk factors. These findings offer insight into the haemodynamic mechanisms by which autonomic dysfunction may contribute to DKD progression and support further investigation into CAN as a target for early intervention strategies to delay or prevent kidney disease.

## Supplementary Information

Below is the link to the electronic supplementary material.

ESM Tables (PDF 38 KB)

## Data Availability

The datasets generated and/or analysed during the current study are not publicly available due to patient confidentiality and institutional data protection policies but are available from the corresponding author on reasonable request, subject to appropriate approvals.

## Abbreviations

ACEi: ACE inhibitor
ARB: Angiotensin II receptor blocker
CAN: Cardiac autonomic neuropathy
CART: Cardiovascular autonomic reflex test
CKD: Chronic kidney disease
DKD: Diabetic kidney disease
GLP-1 RA: Glucagon-like peptide-1 receptor agonist
KDIGO: Kidney Disease: Improving Global Outcomes
MRA: Mineralocorticoid receptor antagonist
RAAS: Renin–angiotensin–aldosterone system
SGLT2i: Sodium–glucose cotransporter 2 inhibitor
UACR: Urinary albumin/creatinine ratio
