# Supplementary material for: Cardiac autonomic neuropathy predicts renal decline in type 1 diabetes mellitus: a 15 year follow-up study
Source: Diabetologia. 2026 Jul 29;69(10):2983–92. doi: 10.1007/s00125-026-06818-y (PMC13541789; doi:10.1007/s00125-026-06818-y)
Supplement: Supplementary file 1 [file 125_2026_6818_MOESM1_ESM.pdf]

Electronic summary material (ESM)

Table 1: Evaluation of Cardiovascular reflex tests by O'Brien protocol

Autonomic function test	Measured parameter	Normal value (score 0)	Limit value (score 1)	Abnormal value (score 2)
Parasympathetic function analysis<br>Heart Rate Response to Deep Breathing	E/I ratio	≥ 1.21	1.10-1.20	< 1.10
Heart Rate Response to the Valsalva Manoeuvre	Valsalva ratio	≥ 1.21	1.11-1.20	< 1.10
Heart Rate Response to Standing (30:15 Ratio):	30:15 ratio	≥ 1.04	1.01-1.03	< 1.00
Sympathetic function analysis<br>Blood Pressure Response to Standing	Systolic blood pressure drop (mmHg)	< 10	10-20	> 20
Blood Pressure Response to Sustained Handgrip	Diastolic blood pressure elevation (mmHg)	≥ 16	11-15	< 10

Table 2: Comparison of estimated glomerular filtration rate (eGFR, ml/min/1.73m2) and urinary albumin creatinine ratio (UACR, mg/mmol) changes in no cardiac autonomic neuropathy (no CAN) and CAN in participants with normal eGFR (≥ 90ml/min/1.73m2, G1) and normoalbuminuria (UACR < 3mg/mmol, A1) using multiple regression analysis (p-value and 95% confidence intervals)

	No CAN (n=46)	CAN (n=23)	Model 1	Model 2	Model 3
eGFR decline	3.3 (7.7)	3.2 (12.2)	0.96 (-4.9:4.7)	0.45 (-3.3:7.4)	0.64 (-6.3:3.9)
UACR increase	0.4 (0.9)	7.0 (20.0)	0.03 (0.7:12.4)	0.09 (-0.8:12.5)	0.03 (1.0:15.5)

Table 3: Summary of follow up estimated glomerular filtration rate (eGFR, ml/min/1.73m2) and urinary albumin creatinine ratio (UACR, mg/mmol) changes in different KDIGO risk groups

	Low Risk (N=77)	Moderate Risk (N=7)	High/Very High Risk (N=3)	P value
eGFR decline (ml/min/1.73m2)	4.1(9.6)	10.2(17.8)	6.7(11.5)	0.30
UACR increase (mg/mmol)	2.8(11.5)	1.8(3.9)	42.9(74.3)	<0.001
